# Clinical application of real-time tumor-tracking for stereotactic volumetric modulated arc therapy for liver tumors

**DOI:** 10.1016/j.phro.2024.100623

**Published:** 2024-08-05

**Authors:** Naoki Miyamoto, Norio Katoh, Takahiro Kanehira, Kohei Yokokawa, Ryusuke Suzuki, Yusuke Uchinami, Hiroshi Taguchi, Daisuke Abo, Hidefumi Aoyama

**Affiliations:** a Faculty of Engineering, Hokkaido University, North13, West 8, Kita-ku, Sapporo, Hokkaido 060-8628, Japan; b Department of Medical Physics, Hokkaido University Hospital, North 14, West 5, Kita-ku, Sapporo, Hokkaido 060-8648, Japan; c Faculty of Medicine, Hokkaido University, North 15, West 7, Kita-ku, Sapporo, Hokkaido 060-8638, Japan; d Department of Radiation Oncology, Hokkaido University Hospital, North 14, West 5, Kita-ku, Sapporo, Hokkaido 060–8648, Japan; e Department of Diagnostic and Interventional Radiology, Hokkaido University Hospital, North 14 West 5, Kita-ku, Sapporo, Hokkaido 060-8648, Japan

**Keywords:** Motion management, SBRT, VMAT, Internal marker, Respiratory gating

## Abstract

Real-time tumor-tracking volumetric modulated arc therapy (RT-VMAT) enabling beam-gating based on continuous X-ray tracking of the three-dimensional position of internal markers is relevant for moving tumors. Dose-volume characteristics and treatment time were evaluated in ten consecutive patients who underwent liver stereotactic body radiation therapy with RT-VMAT. Target dose conformity and sparing of the stomach and the intestine were improved comparing RT-VMAT with RT-3D conformal radiotherapy. The mean treatment time for each fraction was less than 10 min. RT-VMAT could be effective, especially for targets located adjacent to organs at risk.

## Highlights

• Respiratory-gated volumetric arc therapy with internal markers is clinically feasible.
• Mean treatment time for stereotactic liver irradiation was below 10 min.
• Target conformity and stomach-sparing profit from gated volumetric arc therapy.

## 1 Introduction

Respiratory-gated volumetric modulated arc therapy (VMAT) is promising for achieving better dose distribution than three-dimensional (3D) conformal radiotherapy [1], [2], [3]. Furthermore, beam gating based on the internal markers is expected to be superior to that based on the external markers due to a better correlation with the tumor motion [4], [5], [6], [7]. To date, VMAT using triggered kilovoltage (kV) X-ray imaging to monitor the internal marker position during beam-gating based on external surrogates has been reported [8]. A tool using kV X-ray imaging for monitoring the marker positions throughout treatment has been clinically applied [9]. Although real-time tumor-tracking enabling beam-gating based on the three-dimensional position of internal markers had been conducted with 3D conformal radiotherapy [10], [11], [12], [13], [14], no studies have reported its clinical application in VMAT.

In recent years real-time tumor-tracking using internal fiducial markers and kV X-ray imaging to guide gating in stereotactic body radiation therapy (SBRT) has been initiated for VMAT (called RT-VMAT in this study). To the best of our knowledge, this is the first report of its clinical application. In this study, we aimed to show clinical feasibility of RT-VMAT based on dose-volume characteristics and treatment time.

## 2 Materials and methods

### 2.1 Patient characteristics

We evaluated 10 consecutive patients with a single target who underwent liver SBRT with RT-VMAT at our institution between April 2022 and December 2022. The patients were categorized into two groups, the adjacent and non-adjacent groups, based on our institutional protocol [12]. In the non-adjacent group, the tumor was located more than 2 cm away from the intestinal tract and hepatic hilum. In the adjacent group, the tumor was located within 2 cm of the intestinal tract or hepatic hilum. Each group included five patients. A spherical gold marker (iGold, MEDIKIT, Japan) with a diameter of 2 mm was percutaneously implanted near the tumor. The relevant details of the patient characteristics are summarized in Supplementary Table S1.

### 2.2 Treatment system

**Fig. 1:**
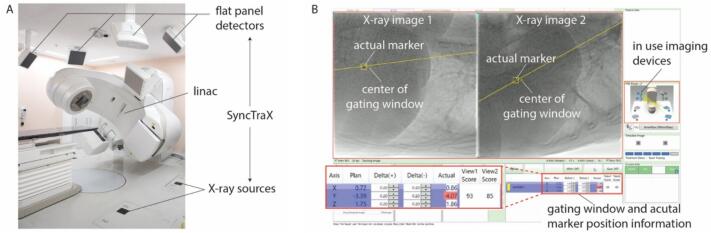
(A) Overview of the treatment system which consists of linac and SyncTraX. (B) Snapshot of the SyncTraX software. Position and tolerance width of the gating window are shown as “Plan” and “Delta (+/-)”, respectively.

All patients were treated using a Linac (TrueBeam; Varian Medical Systems, USA) equipped with a real-time X-ray imaging system (SyncTraX FX4; Shimadzu, Japan). The SyncTraX system (Fig. 1) was used to evaluate the three-dimensional position of internal markers during treatment. It consists of four kV X-ray imaging units. Two X-ray imaging units capable of capturing images without overlapping the gantry head were selected automatically. The X-ray images were obtained at a maximum rate of 30 frames/s. The projected positions of the markers in each X-ray image were automatically recognized using template-pattern matching [15]. Success or failure of image recognition was determined online based on a quantitative score, ranging from 0 to 100, derived from template-pattern matching. The beam irradiation was disabled when the recognition score felt below a threshold value. In our institution, the threshold was empirically set at 30. As the marker recognition performance depends on the type of marker, patient body thickness, marker location, and other factors, it was validated in a phantom test before clinical application [16]. The treatment beam was switched on only when the actual marker position was within the gating window (Fig. 1B). During RT-VMAT, irradiation was performed while rotating intermittently according to the respiratory motion. Because X-ray imaging is blocked by the gantry head if rotational irradiation is continued, gantry angles in the range of $179^{{}^{\circ}}-181^{{}^{\circ}}$, $89^{{}^{\circ}}-96^{{}^{\circ}}$, $3^{{}^{\circ}}-357^{{}^{\circ}}$, and $264^{{}^{\circ}}-271^{{}^{\circ}}$ were not used in RT-VMAT. The system latency for the beam-on and beam-off was approximately 150 and 70 ms, respectively [17].

Before clinical application, dosimetric verification was conducted with an ion chamber and radiochromic film using a dynamic phantom (Supplementary material A).

### 2.3 Treatment planning

Exhaled breath-hold CT images were used for treatment planning. For treatment plan optimization with a 6MV flattening filter free beam a collapsed cone algorithm was used with a 2 mm grid size, a gantry spacing of 2° and a maximum dose rate of 1400 monitor units (MU)/min. Further details are listed in the Supplementary Table S2. The gross tumor volume –clinical target volume (CTV) margin ranged from 0 to 5 mm, depending on the size and location of the tumor and the patient's liver function. The CTV–planning target volume (PTV) margin was 5 mm. The planning organ at-risk volume (PRV) margin was 5 mm. The prescribed doses for the nonadjacent and adjacent groups were 40 Gy in four fractions and 48 Gy in eight fractions for PTV D_95%_, respectively. The dose constraints for the targets and OARs are listed in Supplementary Table S3. In the nonadjacent group, one case with an additional dose constraint of D_max_ < 12 Gy to the lead of the pacemaker and one case with D_mean_ < 3 Gy to the past irradiation area were included. The adjacent group included a patient who required an additional constraint of D_max_ < 4 Gy to the lung owing to a medical history of interstitial pneumonia. Depending on the case, three to six partial arcs were used to compensate for angles that could not be used for RT-VMAT (Supplementary Table S4).

### 2.4 Treatment workflow

The initial patient setup was performed by image registration between the orthogonal kV X-ray images obtained using a gantry-mounted onboard imager and digitally reconstructed radiographs with reference to bony structures. CBCT images were also utilized when the rotational deviations were included. Next, kV X-ray imaging with SyncTraX was initiated and stopped during the expiration respiratory phase. The discrepancy between the actual marker position during expiration and the gating window was derived and applied to the patient couch. If the discrepancy, namely the difference between bony structure-based alignment and marker-based alignment, was not clinically acceptable (e.g., 5 mm in a typical case), it was recommended to acquire the CT or CBCT images to exclude marker migration. It is well known that the markers inserted in lung region often dropout [18]. We did not experience marker dropout in the liver cases assessed in this study. Thus, the treatment beam irradiation was enabled while exhaling during free breathing. The tumor position while exhaling could be altered owing to baseline shift/drift during treatment [19]. When baseline shift/drift was observed by visual inspection of the X-ray images, X-ray imaging was stopped during exhalation and couch position was corrected as in the initial patient setup process. A gating window width of ± 2 mm in each direction was applied in all patients.

### 2.5 Evaluation and statistical analysis

For comparison, treatment plans for real-time tumor-tracking 3D conformal radiotherapy (RT-3D) with nine coplanar beams were created retrospectively by an independent physicist without knowledge of the VMAT plan. RT-3D plans were created to satisfy the target doses and meet the criteria for the OAR dose as much as possible. Wilcoxon signed-rank sum test was used to test the statistical significance of median values of each dose index.

The cumulative imaging time of kV X-ray for each fraction were evaluated using the recorded RT-VMAT data. Treatment time was defined as the time from the first X-ray imaging to the last of treatment beam irradiation. The gating efficiency was defined as the ratio of the gate-on time to the imaging time. The imaging time in RT-3D was estimated by tracing the time-series data of the gate-on/off status, assuming a fixed dose rate of 1400 MU/min. The treatment time in RT-3D was estimated as the sum of the imaging time and interval time between the nine beams. The interval time, defined as the time from the last X-ray imaging to the first imaging in the next field, was estimated from the recorded data to be approximately 28 s. Hence, the total interval time for RT-3D with nine coplanar beams was assumed to be 28×8=224 s.

## 3 Results

### 3.1 Dosimetric evaluation

**Fig. 2:**
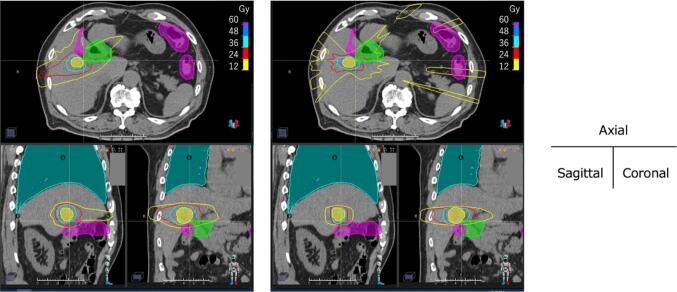
Example of dose distributions of (left) RT-VMAT and (right) RT-3D. The ROIs represent the PTV (yellow), duodenum PRV (green), intestine PRV (magenta), and lung (cyan), respectively. The dose constraint for duodenum PRV and intestine PRV was D_max_ < 36 Gy. (For interpretation of the references to colour in this figure legend, the reader is referred to the web version of this article.)

Relatively large difference was found in median dose for the stomach in the non-adjacent group. D_0.5cc_ for stomach in RT-VMAT and RT-3D were 3.5 (2.2–11.7) Gy and 10.7 (2.3–16.9) Gy, respectively. As the direction of the beam was limited for two atypical cases, including additional dose constraints to consider the pacemaker cable and past irradiation for each, a relatively high dose was delivered to the stomach in RT-3D. In the adjacent group, median dose of D_0.5cc_ for intestine was tended to be significantly smaller in RT-VMAT. Details of the dose indices are summarized in Supplementary Table S3. Fig. 2 shows an example of dose distribution in a patient with a medical history of interstitial pneumonia. In RT-3D, as most beams had to be arranged in the lateral direction to spare the lungs, it was difficult to control the high-dose region to the adjacent OARs while maintaining the prescribed doses to the target, and the two-dose constraints for the lung and duodenum PRV were not met. There was no significant difference in MU values between RT-VMAT and RT-3D in either group.

### 3.2 Imaging time, treatment time, and gating efficiency

In the nonadjacent group, the mean ± SD of imaging time in RT-VMAT and RT-3D were 5.7 ± 2.9 min and 4.8 ± 1.9 min, respectively. Treatment time were 7.5 ± 3.9 min and 8.5 ± 1.9 min, respectively. In the adjacent group, the mean ± SD of imaging time in RT-VMAT and RT-3D were 7.4 ± 2.1 min and 3.7 ± 0.7 min, respectively. Treatment time were 9.6 ± 2.7 min and 7.4 ± 0.7 min, respectively. The imaging and treatment times of RT-VMAT in the adjacent group were longer than those of RT-3D because the dose rate in RT-VMAT was highly modulated to form a complex dose distribution.

All patients were treated without frequent marker recognition failure. The mean score and successful rate of image recognition in daily treatment for ten patients were 79.0 ± 7.6 and 98.8 ± 1.3 %, respectively. The average number, SD and range of baseline shift/drift correction in one fraction was 0.7 ± 1.0 (0–5). Gating efficiency in the nonadjacent and adjacent groups were 34.0 ± 9.6 % and 28.8 ± 4.7 %, respectively.

## 4 Discussion

In this study, RT-VMAT satisfied most of the dose constraints in all patients, including atypical cases. The target dose may have had to be lowered to spare the OARs if RT-3D had been applied in some cases. In addition, RT-VMAT can be applied to patients with multiple targets, who were excluded from the evaluation in this study because of the difficulty in comparing them with RT-3D. In both RT-VMAT and RT-3D, noncoplanar beams may result in better dose distribution [20], although the treatment time could be prolonged [21].

External surrogates such as an external marker block are used for respiratory beam gating as standard technique at the present time [22], [23], [24], [25]. However, the motion correlation between the internal markers and the target is higher than that between the external surrogate and the target [4], [5], [6], [7]. Therefore, ITV and PTV margins for RT-VMAT could be smaller than that for standard respiratory gating techniques.

One limitation of RT-VMAT is the dose required for X-ray imaging. Assuming the typical X-ray imaging condition of 100 kV, 80 mA, 3 msec pulse width and 15 frames/s for liver SBRT, the accumulated skin dose is estimated to be approximately 0.2 Gy for one hour. Although an additional imaging dose does not induce severe skin reactions, the X-ray tube voltage and current should be minimized while maintaining stable marker recognition. In this study, one marker was used as surrogate. However, deformations or rotations may be included. If multiple markers can be implanted surrounding the tumor, the target localization accuracy will be enhanced [26].

In conclusion, RT-VMAT could be clinically feasible and effective, especially in cases requiring complex dose distributions, such as multiple targets and targets adjacent to OARs.

## CRediT authorship contribution statement

**Naoki Miyamoto:** Conceptualization, Methodology, Investigation, Formal analysis, Writing – original draft. **Norio Katoh:** Methodology, Investigation, Writing – review & editing. **Takahiro Kanehira:** Formal analysis. **Kohei Yokokawa:** Formal analysis. **Ryusuke Suzuki:** Formal analysis. **Yusuke Uchinami:** Writing – review & editing. **Hiroshi Taguchi:** Writing – review & editing. **Daisuke Abo:** Writing – review & editing. **Hidefumi Aoyama:** Writing – review & editing.

## Declaration of Competing Interest

The authors declare that they have no known competing financial interests or personal relationships that could have appeared to influence the work reported in this paper.
