# Supplementary material for: Clinical application of real-time tumor-tracking for stereotactic volumetric modulated arc therapy for liver tumors
Source: Phys Imaging Radiat Oncol. 2024 Aug 5;31:100623. doi: 10.1016/j.phro.2024.100623 (PMC11367098; doi:10.1016/j.phro.2024.100623)
Supplement: Supplementary Data 1 [file mmc1.docx]

**Supplementary Materials**

Table S1: Patient characteristics. The range of respiratory motion was evaluated as the 95% range of marker displacement during a treatment day in the left–right (LR), superior–inferior (SI), and anterior–posterior (AP) direction. Distance between the GTV and marker was evaluated as three-dimensional length between the marker and the center of gravity of the GTV.

|  | | Nonadjacent group  40 Gy/4fr | Adjacent group  48 Gy/8fr |
| --- | --- | --- | --- |
| Number of patients (male/female) | | 2/3 | 4/1 |
| Age (median and range) [years] | | 83 (75–90) | 74 (56–78) |
| GTV volume (median and range) [cm^3^] | | 8.6 (0.2–61.1) | 12.1 (4.7–17.8) |
| Equivalent sphere diameter of GTV  (median and range) [mm] | | 25.4 (7.6–48.9) | 28.5 (20.8–32.4) |
| PTV volume (median and range) [cm^3^] | | 55.6 (8.8–124.2) | 34.1 (17.9–90.6) |
| Equivalent sphere diameter of PTV  (median and range) [mm] | | 47.3 (25.6–61.9) | 40.2 (32.5–55.7) |
| Normal liver (liver-GTV) volume  (median and range) [cm^3^] | | 945.4 (705.5–1339.1) | 1382.3 (1219.6–1495.8) |
| Distance between the GTV and marker (median and range) [mm] | | 47.7 (14.9–70.6) | 57.5 (31.4–67.9) |
| Range of respiratory motion  (mean ± SD) [mm] | LR | 2.9 ± 1.7 | 3.3 ± 3.0 |
|  | SI | 14.0 ± 6.5 | 15.2 ± 4.5 |
|  | AP | 5.1 ± 1.9 | 5.8 ± 1.2 |

Table S2: Basic setting for treatment planning

| CT scanner | SOMATOM (Siemens, Germany) |
| --- | --- |
| Image resolution | 0.98 mm/pixel |
| Slice thickness | 2 mm |
| Image acquisition setup | arms up with vacuum cushion |
| Treatment planning system | RayStation (version 10.0.1) |
| Dose calculation algorithm | collapsed cone convolution |
| Calculation grid size | 2 mm |
| Gantry spacing in dose calculation | 2° |
| Beam type | 6 MV flattening filter free |
| Maximum dose rate | 1400 monitor unit/min |

| Table S3: Dose–volume constraints and comparison of median (minimum – maximum) values for targets and OARs in nonadjacent (40 Gy) and adjacent (48 Gy) group. The number shown as the upper-right subscript represents the number of cases in which the constraint was not met. | | | | | | | | |
| --- | --- | --- | --- | --- | --- | --- | --- | --- |
|  |  | Nonadjacent group  40 Gy/4fr (n = 5) | |  | Adjacent group  48 Gy/8fr (n = 5) | |  |  |
|  | Dose volume constraints for 40/48 Gy group | RT-VMAT | RT-3D | *p* | RT-VMAT | RT-3D | *p* |  |
| PTV | D_95%_ (Gy) ≧ 40/48 | 40.0  (40.0 - 40.0) | 40.3  (40.0 - 41.0) | 0.25 | 48.0  (48.0 - 48.0) | 48.3  (48.0 - 48.7) | 0.25 |  |
|  | D_50%_ (Gy) ≧ 45/54 | 45.7  (45.4 - 49.7) | 47.5  (46.7 - 48.6) | 0.44 | 55.7  (55.0 - 57.3) | 57.0  (56.8 - 58.2) | 0.13 |  |
| GTV | D_2%_ (Gy) ≧ 50/60 | 51.4  (50.1 - 53.3) | 50.4  (50 - 50.6) | 0.13 | 60.6  (60.2 - 62.7) | 60.4  (60.1 - 62.3) | 0.63 |  |
|  | D_max_ (Gy) ≦ 52.5/63 | 51.6  (50.7 - 53.9) | 50.5  (50.1 - 51.0) | 0.06 | 60.8  (60.7 - 63.0) | 60.8  (60.3 - 62.7) | 0.38 |  |
| Liver-GTV | V_<17.5Gy_ (cc) ≧ 700 cm^3^ | — | — | — | 1206.67  (863.34 - 1357.31) | 1199.96 (804.21 - 1307.43) | 0.44 |  |
|  | V_23.6Gy_ (%) ≦ 20％ | — | — | — | 8.5  (4.2 - 18.3) | 8.5  (5.5 - 21.4)^(1)*^ | 0.69 |  |
|  | V_15Gy_ (%) ≦ 30％ | — | — | — | 23.1 (13.2 - 29.3) | 22.7  (12.3 - 39.8)^(1)*^ | 0.31 |  |
|  | V_10Gy_ (%) ≦ 50％ | — | — | — | 35.3  (26.5 - 45.9) | 38  (23.2 - 46.6) | 1.00 |  |
|  | V_<15Gy_ (cc) ≧ 700 cm^3^ | 736.67  (604.41 - 1024.08)^(2)*^ | 668.63 (610.52 - 1058.76)^(3)*^ | 0.63 | — | — | — |  |
|  | V_18Gy_ (%) ≦ 20％ | 12.4  (4.2 - 17.9) | 12.2  (4.3 - 20.1)^(1)*^ | 0.88 | — | — | — |  |
|  | V_12Gy_ (%)≦ 30％ | 20.2  (7.6 - 28.8) | 17.2  (7.7 - 46.2)^(2)*^ | 0.63 | — | — | — |  |
|  | V_8Gy_ (%)≦ 50％ | 27.3  (12.6 - 45.4) | 24.8  (14.2 - 64.7)^(1)*^ | 0.50 | — | — | — |  |
| Duodenum | D_max_ (Gy) ≦ 27/35 | 8.0  (0.4 - 21.4) | 11.7  (0.5 - 21.7) | 0.25 | 18.95  (1.4 - 28.2) | 16.45  (1.4 - 29.7) | 1.00 |  |
|  | D_0.5cc_ (Gy) ≦ 24/32 | 9.0  (0.4 - 17.4) | 15.6  (0.4 - 21.4) | 0.50 | 13.9  (1.2 - 22.3) | 12.4  (1.2 - 22.5) | 0.75 |  |
| Duodenum PRV | D_max_ (Gy) ≦ 29/36 | 13.0  (0.5 - 26) | 18.6  (0.6 - 28.6) | 0.25 | 31.65  (1.6 - 35.4) | 34.5  (1.7 - 40.9)^(1)*^ | 0.38 |  |
|  | D_0.5cc_ (Gy) ≦ 27/33.6 | 10.2  (0.5 - 22.4) | 16.1  (0.5 - 21.9) | 1.00 | 23.1  (1.4 - 29.5) | 23.45  (1.5 - 29.8) | 0.63 |  |
| Stomach | D_max_ (Gy) ≦ 27/36 | 3.9  (2.7 - 13.2) | 11.1  (4.8 - 21.5) | 0.06 | 15.8  (11.7 - 21.7) | 16.0  (3.7 - 23.4) | 0.81 |  |
|  | D_0.5cc_ (Gy) ≦ 24/33.6 | 3.5  (2.2 - 11.7) | 10.7  (2.3 - 16.9) | 0.19 | 13.6  (10.3 - 19.9) | 14.9  (3.2 - 22.5) | 0.81 |  |
| Stomach PRV | D_max_ (Gy) ≦ 29/39 | 9.4  (3.7 - 14.6) | 15.15  (6.7 - 21.8) | 0.13 | 18.6  (12.3 - 22.1) | 16.9  (11.6 - 28.1) | 1.00 |  |
|  | D_0.5cc_ (Gy) ≦ 27/36 | 8.3  (3.4 - 13.2) | 14.1  (5.8 - 21.5) | 0.13 | 16.7  (11.4 - 21.3) | 16.2  (5.5 - 23.4) | 0.63 |  |
| Intestine | D_max_ (Gy) ≦ 27/35 | 5.8  (2.4 - 15.9) | 17.2  (2.0 - 17.8) | 0.50 | 15.4  (0.8 - 22.2) | 15.6  (0.9 - 25.8) | 0.31 |  |
|  | D_0.5cc_ (Gy) ≦ 24/32 | 6.2  (2.1 - 11.4) | 16.2  (1.7 - 18.4) | 0.50 | 13.9  (0.7 - 17.4) | 15.1  (0.8 - 23.6) | 0.06 |  |
| Intestine PRV | D_max_ (Gy) ≦ 29/36 | 6.9  (2.6 - 19.2) | 17.9  (2.1 - 18.9) | 1.00 | 17.3  (1.0 - 34.2) | 23.9  (1.1 - 34.2) | 0.31 |  |
|  | D_0.5cc_ (Gy) ≦ 27/33.6 | 6.4  (2.4 - 16.6) | 17.2  (2.0 - 18.4) | 0.50 | 15.5  (0.9 - 26.4) | 15.7  (0.9 - 28.4) | 0.75 |  |
| Spinal cord PRV | D_max_ (Gy) ≦ 25/33.4 | 6.0  (4.9 - 18) | 7.6  (3.7 - 13.5) | 0.31 | 5.3  (2.9 - 18.9) | 14.8  (4.7 - 16.8) | 0.81 |  |
|  | D_0.35cc_ (Gy) ≦ 20/26 | 5.3  (4.4 - 14.1) | 6.5  (2.9 - 12.6) | 0.44 | 5.0  (2.6 - 17.3) | 13.9  (4.6 - 16.5) | 0.31 |  |
|  | D_1.2cc_ (Gy) ≦ 13.2/16.8 | 4.9  (4.0 - 12.7) | 5.4  (2.2 - 12.0) | 0.38 | 4.8  (2.4 - 16.6) | 15.4  (6.7 - 45.0) | 0.19 |  |
| CI |  | 1.19  (1.10 - 1.25) | 1.30  (1.15 - 1.37) | 0.06 | 1.17  (1.07 - 1.34) | 1.34  (1.16 - 1.36) | 0.06 |  |
| * Two patients whose normal liver volume was approximately 700 cc were included. | | | | | | | | |

| Table S4: Range of gantry angles for RT-VMAT including rotation direction, collimator angle and MU values for each dose prescription. All beams were arranged to be coplanar beams with 0 degrees of couch rotation. | | | | | | |
| --- | --- | --- | --- | --- | --- | --- |
| Nonadjacent group  (40 Gy/4fr) | | |  | Adjacent group  (48 Gy/8fr) | | |
| Patient | # of arcs | Angle range [deg]  (rotation direction, collimator angle [deg], MU values) |  | Patient | # of arcs | Angle range [deg]  (rotation direction, collimator angle [deg], MU values) |
| 1 | 4 | 181–264 (CW, 10, 624.24)  271–357 (CW, 10, 370.97)  357–271 (CCW, 350, 492.70)  264–181 (CCW, 350, 596.99) |  | 1 | 6 | 240–264 (CW, 10, 169.69)  271–357 (CW, 10, 276.62)  3–70 (CW, 10, 212.77)  70–3 (CCW, 350, 210.91)  357–271 (CCW, 350, 253.21)  264–240 (CCW, 350, 57.19) |
| 2 | 3 | 35-5 (CCW, 10, 532.27)  355–295 (CCW, 10, 940.71)  260–210 (CCW, 10, 693.96) |  | 2 | 4 | 179–96 (CCW, 90, 459.74)  89–3 (CCW, 90, 400.44)  357–271 (CCW, 90, 320.87)  264–181 (CCW, 90, 429.29) |
| 3 | 3 | 179–141 (CW, 10, 710.69)  295–271 (CW, 10, 560.48)  263–181 (CW, 10, 1207.75) |  | 3 | 4 | 134–96 (CCW, 45, 316.68)  89–3 (CCW, 45, 546.86)  357–271 (CCW, 45, 477.52)  264–226 (CCW, 45, 266.69) |
| 4 | 4 | 181–264 (CW, 10, 467.46)  271–340 (CW, 10, 412.58)  340–271 (CCW, 350, 464.27)  264–181 (CCW, 350, 402.06) |  | 4 | 4 | 181–264 (CW, 10, 334.22)  271–357 (CW, 10, 299.16)  357–271 (CCW, 350, 312.40)  264–181 (CCW, 350, 310.99) |
| 5 | 3 | 35-3 (CCW, 10, 476.07)  357–271 (CCW, 10, 802.59)  264–240 (CCW, 10, 390.14) |  | 5 | 4 | 3–89 (CW, 10, 617.71)  96–179 (CW, 10, 422.48)  179–96 (CCW, 350, 459.74)  264–181 (CCW, 350, 307.08)  181–264 (CW, 10, 268.16) |

**Appendix A: Dosimetric verification with ion-chamber and radiochromic film**

As one of the commissioning tests for clinical use, dosimetric verification was performed. A simulated RT-VMAT plan of which dose prescription was 40 Gy/4 fr was created using previous liver SBRT case. Then, CT images were replaced with a quality assurance (QA) phantom (I'mRT phantom, IBA dosimetry, Germany) and reference dose was obtained by forward calculation. By using an in-house motion platform which enables to reproduce actual respiratory motion in SI direction, the QA phantom containing markers inside was moved and irradiated with RT-VMAT. The absolute dose at the target center was measured with an ionization chamber and compared with the planned dose. In addition, the two-dimensional dose distribution in the coronal plane of the target center was obtained by Gafchromic EBT3 Film (Ashland, USA) and verified with planned dose by gamma analysis. Gamma value was evaluated in the region of where the dose was more than 10% of the maximum dose in planned dose, and 2mm/2%, 2mm/3%, 3mm/2%, and 3mm/3% were used as the distance and dose criteria in the gamma analysis. RIT113 (Radiological Imaging Technology, USA) was used for gamma analysis.

In the ionization chamber measurement, the dosimetric error between plan and actual was within 3%. The planned and measured two-dimensional dose distributions in the coronal plane, gamma value distribution, and dose profiles are shown in Figure S1. Motion direction was corresponding to superior-inferior (SI) direction. As shown in Figure S1(e), the penumbra of the profile at the target edge was slightly expanded. This dosimetric blur could be compensated by an appropriate margin. The gamma pass rates at 2mm/2%, 2mm/3%, 3mm/2%, and 3mm/3% were 93.2%, 98.7%, 98.6%, and 99.8%, respectively.


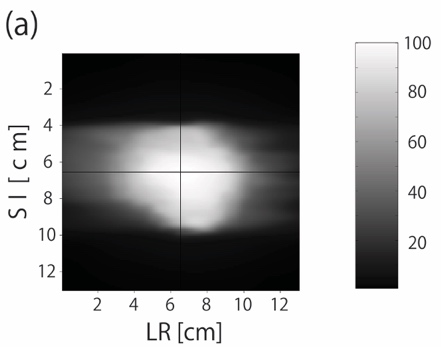

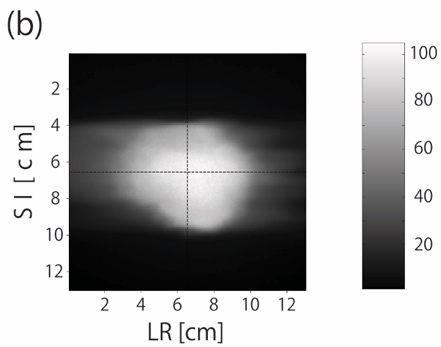

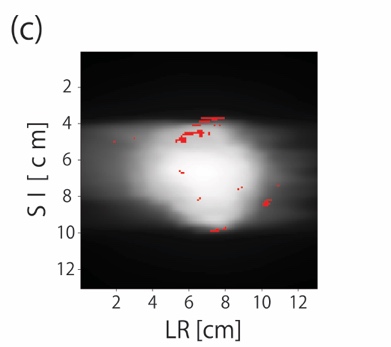


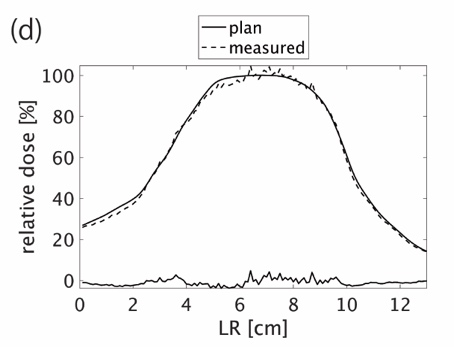

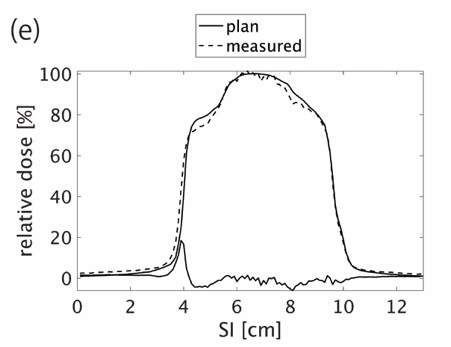


Figure S1: (a) Planned and (b) measured dose distribution on coronal plane. (c) The result of gamma analysis with 2mm/3% criteria. The red-colored filled area represents the region where gamma value was greater than 1. Dose profile along to (d) LR and (e) SI direction.
